# Heat Stroke Warning System Prototype for Athletes: A Pilot Study

**DOI:** 10.3390/s25020294

**Published:** 2025-01-07

**Authors:** Kanchana Silawarawet, Phattarakorn Kaewchukul, Sairag Saadprai

**Affiliations:** 1 Department of Electrical and Computer Engineering, Faculty of Engineering, Thammasat School of Engineering, Thammasat University, Pathumthani 12120, Thailand; skanchan@engr.tu.ac.th (K.S.); kphattarakorn@gmail.com (P.K.); 2 Department of Sports Science and Sports Development, Faculty of Allied Health Sciences, Thammasat University, Pathumthani 12120, Thailand

**Keywords:** heat stroke, sensor, IoT, heat index, athletes

## Abstract

This research has developed a heat stroke warning system prototype for athletes utilizing the following sensors: DHT22, GY-906-BAA MLX90614, MAX30102. The device calculates the heat stroke risk and notifies users. The data is recorded, stored, displayed on a free-access website which graphs body temperature, ambient temperature, humidity, heart rate and heat stroke risk, and provides notifications for athletes engaged in outdoor activities. The researchers recorded sensors data (*n* = 1) for two sessions (12 min/session) in a closed room, at the sixth-minute marker, with an air conditioner activated to observe the changes observed by the sensors. For accuracy, the researchers employed Criterion-Related Validity, comparing sensor against standard equipment measurement. For reliability, we utilized Test-Retest Reliability, comparing sensor data from the first and second measurements. Accuracy and reliability were evaluated using the Pearson Correlation Coefficient, with significance set at *p* < 0.01. The DHT22 sensor demonstrates very high accuracy (r = 0.923) in ambient temperature and (r = 0.774) humidity measurements. It showed no significant reliability (r = 0.489) in temperature and (r = 0.185) humidity measurements. The GY-906-BAA MLX90614 sensor exhibited very high accuracy (r = 0.923) and reliability (r = 0.866) in body temperature measurements. The MAX30102 sensor lacked significant accuracy (r = 0.179) and reliability (r = 0.171) in heart rate measurements. The development of accuracy and reliability of sensors are important for preventing heat stroke in future applications.

## 1. Introduction

Heat stroke is a life-threatening condition that occurs when the body overheats due to prolonged exposure to high temperatures or physical exertion. With a body temperature of 40 degrees Celsius or more associated with multiorgan dysfunction [1], heat stroke can lead to severe complications affecting vital organs such as the brain, heart, lungs, kidneys, and muscles [1,2]. Moreover, vulnerable groups, such as the elderly, children, infants, athletes, individuals engaging in activities like jungle trekking, military training, outdoor workers, and those with pre-existing health conditions, are at higher risk. Heat stroke can lead to death through various mechanisms, including multi-organ failure and sudden cardiac arrest. Predisposing conditions like dehydration, cardiovascular diseases, high levels of blood alcohol concentration, and lack of acclimatization to heat can exacerbate the risk [2]. In prolonged instances of hyperthermia, damage to the cerebellum is often prominent, primarily because Purkinje cells are particularly vulnerable to heat-related injury. A core body temperature reaching 40 °C or higher can lead to either lasting or irreversible neurological impairment, aligning with the cellular alterations and cell death that typically occur at these elevated temperatures [3]. These findings emphasize the critical need for early detection and preventive measures, especially among athletes.

There has been significant research on heat stroke detection devices. For instance, P. O. Antonio et al. (2017) [4] developed a heat stroke detection system based on IoT. The system consists of an Arduino Mini 05, the ESP8266 module, a DS18B20 sensor, and a pulse sensor. When changes in body temperature and heart rate reach predetermined conditions, an LED on the Arduino turns on, and a Short Message Service (SMS) is sent to a phone. The advantage of this system is comfort and convenience, as all components are attached to a hand glove, and it provides real-time alerts via SMS. However, a disadvantage is the lack of comprehensive data collection, as it does not measure ambient temperature and humidity, which are crucial for accurately assessing heat stroke risk.

Chen, Lin, Lan, and Hsu (2017) [5] developed a wearable heat-stroke-detection device (WHDD) that uses sensors to monitor physiological parameters such as galvanic skin response, heart rate, and body temperature. This device employs fuzzy logic to evaluate the risk of heat stroke and provides early warnings to users with an alert function. The advantage of this system is its ability to integrate multiple physiological signals for a comprehensive risk assessment. However, its reliance on Bluetooth communication limits its range, making it less effective for outdoor activities where long-distance monitoring is required.

Additionally, Son, Ramli, and Aziz (2021) [6] developed a wearable IoT-based device designed to detect heat stroke. This device utilizes multiple sensors to measure heart rate, ambient temperature, relative humidity, and core body temperature. It calculates the risk of heat stroke using a fuzzy controller and alerts the user through an integrated alert module. The IoT functionality includes a ThingSpeak server and an Android application, which store, visualize, and display physiological data both numerically and graphically. However, using only an Android application limits accessibility, as it cannot be used on other platforms like iOS or PC, making it less versatile compared to a web-based application.

The study involves developing and testing the accuracy and reliability of a heat stroke detection device prototype that integrates sensors capable of monitoring environmental conditions and physiological parameters. Specifically, the device includes an ambient temperature and humidity sensor, an infrared temperature sensor, a heart rate sensor, and a microcontroller. The device calculates the heat index and triggers alerts when it exceeds a specified threshold. Additionally, a web-based monitoring dashboard is created to display trends in graphs and heat stroke risk levels, offering personalized recommendations for heat stroke prevention. These components are used to monitor an athlete’s well-being, focusing on early heat stroke prevention by using the heat index to calculate risk levels. This is only a pilot test of the sensors to demonstrate their feasibility and safety.

## 2. Materials and Methods

### 2.1. Heat Stroke Warning System Design

**Figure 1:**
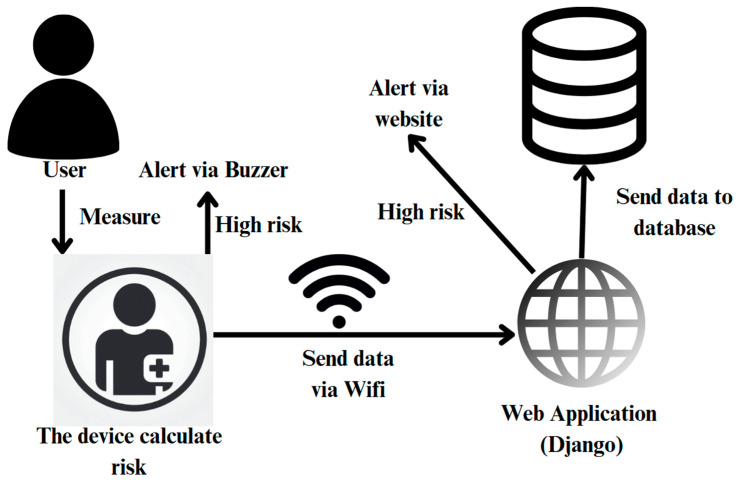
System design overview.

This research designed and implemented a system that combined a hardware device and a software website. One of the researchers measured his heart rate, body temperature, relative humidity, and ambient temperature using the device consisting of the ESP32 microcontroller (Espressif Systems, Shanghai, China), DHT22 ambient temperature (Adafruit, Brooklyn, NY, USA) and relative humidity sensor, MAX30102 pulse oximeter and heart rate sensor (Maxim Integrated, San Jose, CA, USA), active buzzer, and GY-906-BAA MLX90614 infrared temperature sensor (Melexis, Ypres, Belgium). The device used the measured values to calculate the heat stroke risk. Additionally, the device calculated the risk level and if it reached a certain threshold, it notified the users through the buzzer. The device sends the measured data and risk level to the website, developed using the Django framework, via Wi-Fi. The data will then be collected into a database, displayed on the website, and users will be notified through the website when the data indicates a high risk; the system design overview is shown in Figure 1. The researchers utilized and integrated the example code from these open-source codes [7,8,9,10,11,12].

**Figure 2:**
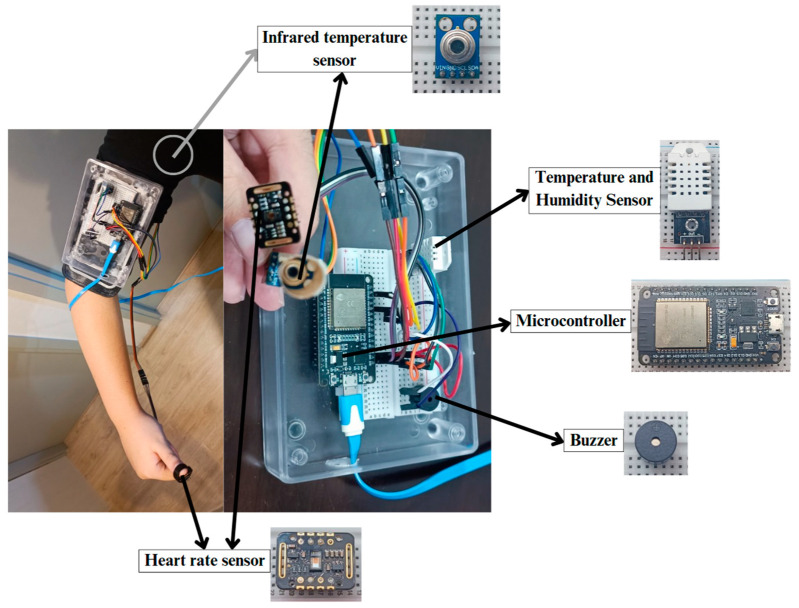
The device components.

The device consists of an ESP32, DHT22, MAX30102 attached on the left thumb fingertip, active buzzer, and GY-906-BAA MLX90614 measured at the left axillary, with a 4 cm soft and elastic tube directed towards the axillary for enhanced accuracy. The researchers chose to measure core body temperature at the axillary for several reasons. Firstly, this method is comfortable for users. Additionally, the axillary is considered a reliable site for assessing core body temperature [13]. Core body temperature can be measured in different ways, such as through the rectum, intestines, or esophagus; additionally, skin temperature at the axillary can indicate core body temperature [14]. The infrared temperature sensor and digital thermometer were used to measure the temperature at the left axillary. The researchers chose not to use rectal measurements because these are impractical, making axillary measurements a better option for this study. However, measuring temperature at the axillary might be difficult during exercise. The device can measure the following values: heart rate, ambient temperature, body temperature, and relative humidity, and can calculate the heat stroke risk. The device’s size is 12 × 10 cm and will be attached to the left of upper arm using an armband. The weight of the device is approximately 250 g. Measured data can be sent to the database using a Hypertext Transfer Protocol (HTTP) Client. The data will be sent in JavaScript Object Notation (JSON) format. The device provides sound notifications via a buzzer speaker at two levels: high risk and very high risk. When the user is ready, they can connect the device to the power bank, which will automatically power on as shown in Figure 2.

The selection of sensors for the developed device is informed by their market availability and the robust support provided by existing technical research. Specifically, we have chosen the ESP32 [15], DHT22 [6], MAX30102 [16,17], and GY-906-BAA MLX90614 [5] due to their proven performance and reliability in similar applications.

### 2.2. Heat Stroke Risk Calculations

To calculate risk, this research has adapted heart rate input table data from Chen, Lin, Lan, and Hsu (2017) [5] and adapted body temperature input according to Balli, Sharan, and Shumway (2023) [18]; body temperature input data are lower than that of Chen, Lin, Lan, and Hsu (2017) [5] for the earlier detection of heat stroke because this purpose of this study is to develop a heat stroke prevention device. Therefore, the researchers used the lower input data; the risk calculation is shown in Table 1 and there are several other studies which are also using similar body temperature thresholds [5,6]. However, this criterion is slightly lower than those established in other research due to safety considerations.

In addition, the thermal heat stroke risk coefficient from Chen, Lin, Lan, and Hsu (2017) [5] has been adjusted to the heat index to suit tropical weather conditions with high temperatures and relative humidity. In this research, the researchers used the heat index equation from Awasthi, Vishwakarma, and Pattnayak (2021) [19] as shown in Equation (1), and created a heat index table as shown in Table 2.

(1) $$\begin{array}{ll} HI=-42.379+ & 2.04901523\times T+10.1433127\times RH-0.22475541\times T\times RH-6.83783\times 10^{-2}\times T^{2}\\ & -5.481717\times 10^{-2}\times {RH}^{2}+1.2287410^{3}\times T^{2}\times RH+8.528210^{-4}\times T\times {RH}^{2}\\ & -1.99\times 10^{-6}\times T^{2}\times {RH}^{2} \end{array}$$

Using the ranges of heat indices from Awasthi, Vishwakarma, and Pattnayak (2021) [19] to create heat stroke risk levels, as shown in Table 3, which has other research following this heat index threshold [20].

Combine the heat stroke risk values from Table 1 and Table 3 and analyze the heat stroke risk as shown in Table 4.

The device sends data and risk data from the sensors to the website and stores it in the database. The website uses the data to create graphs to show the information. When the database receives information that there is a risk, there will be a notification displayed on the website and the device. On the device, there will be a sound through a buzzer that will notify the users if the status is high or very high. The data is reviewed by authorized personnel such as coaches, field medics, or designated health professionals who can have the authority to issue warnings and take necessary actions based on the risk assessments throughout use.

### 2.3. Notification Flowchart

**Figure 3:**
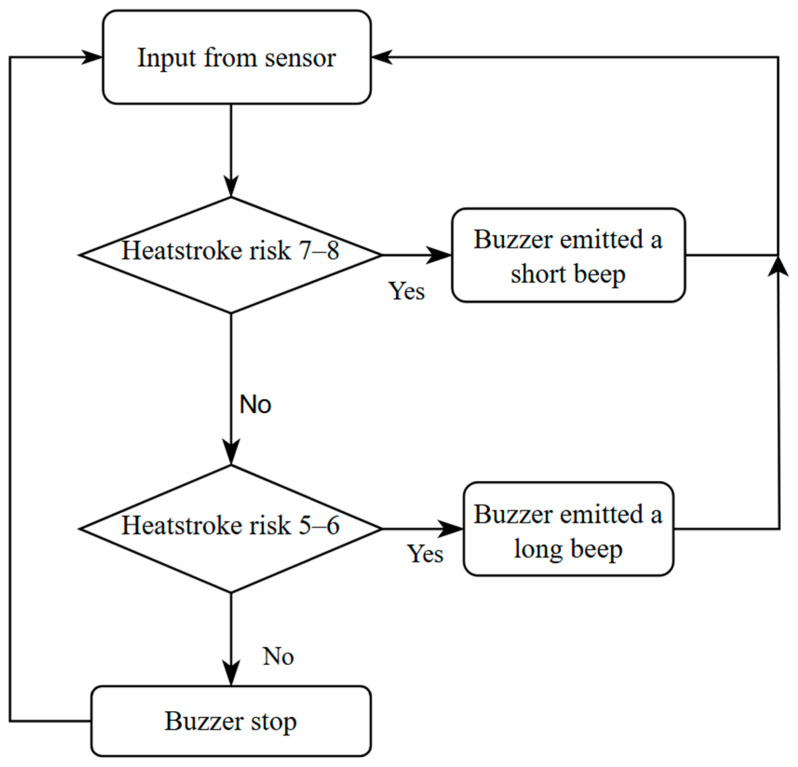
Notification flowchart.

When the device gets the value from the sensors, the device calculates the risk value. If the risk value is from 7 to 8, the buzzer emits a short sound and stop continuously until the risk value decreases. When the risk value is from 5 to 6, the buzzer makes a long sound and stop until the risk value decreases. The buzzer stops when the risk value drops below 5, as shown in Figure 3.

### 2.4. Sitemap

**Figure 4:**
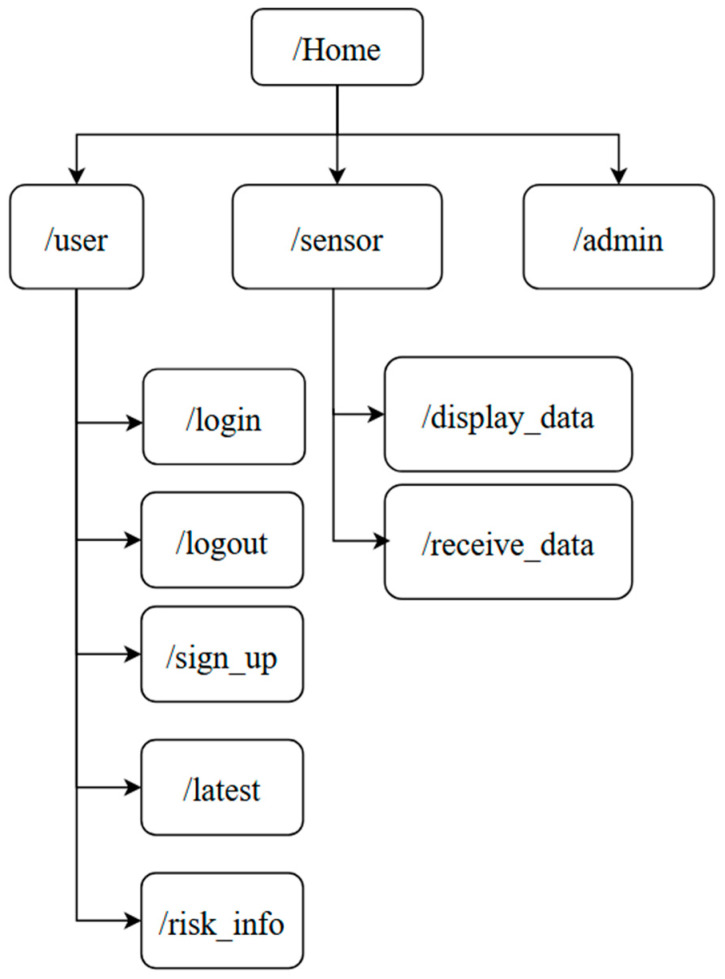
Website sitemap.

When a user visits the website, they first encounter the home page with the information about the project and links to all pages except the admin page. Non-logged-in users can access the login, sign-up, and risk info pages. The sign-up page allows users to register and log in using the login page. The risk info page provides details about the risk status calculation table and guidance on what to do when a risk is detected. Logged-in users can explore the latest page, which displays their most recent data. On the display data page, receives data is presented through graphs showing total and average values. Finally, the received page serves as an Application Programming Interface (API) for receiving data from the device, while the admin page allows viewing of all stored data on the website, as shown in Figure 4.

### 2.5. Testing Procedures

In the sensor’s calibration process, one of the researchers recorded sensor data (one healthy male, aged 22 years old, weight 90 kg, height 173 cm) for two sessions lasting 12 min each. This duration was chosen based on guidelines from the CDC (Centers for Disease Control and Prevention, 2020) [21], which states that heat stroke can develop rapidly, with the body temperature rising to 40.6 °C or higher within 10 to 15 min. The 12-min duration was selected to capture the critical period during which heat stroke symptoms typically manifest, ensuring that the calibration process accurately reflects the conditions under which heat stroke occurs. This is only a pilot test of the sensors to demonstrate their feasibility and safety. However, in the future, researchers plan to collect data from athletes while they are exercising outdoors for longer periods. The location for research collection is at Faculty of Engineering, Thammasat School of Engineering, Thammasat University, Laboratory and Research Building. The testing procedure was held on 30 April 2024 at 2–3 PM. It is important to note that no exercise was involved during this calibration session. The accuracy of the sensor is calculated using Criterion-Related Validity which involves comparing the sensor data with readings from standard equipment and the reliability of the sensor was calculated using Test-Retest Reliability which compares the sensor data with readings from the first and second measurements.

The Criterion-Related Validity and Test-Retest Reliability were assessed using the Pearson Correlation Coefficient. If the correlation coefficient (r) falls between 0.00 and 0.50, the sensor’s accuracy and reliability is considered low. A coefficient between 0.50 and 0.74 indicates medium accuracy and reliability, while a range of 0.75 to 0.90 signifies high accuracy and reliability. An r value between 0.90 and 1.00 denotes very high accuracy and reliability (Portney, 2020) [22] at the confidence interval of 95% and statistical significance was set at *p* < 0.01, Sig. (two-tailed). In cases where the data distribution is not normal, the Spearman Rank Test will be employed as an alternative method to validate the sensor’s accuracy and reliability.

Additionally, the research utilized percentage error to ensure sensor accuracy further, aiming to calibrate the sensor so that the percentage error does not exceed 5.00% [23]. The researchers calculated the percentage error using Equation (2) [23], indicating that if the statistical error is below 5 percent, the measurement errors from the developed device are acceptable when compared to those obtained from the standard device measurements.

(2) $$\%Error=\frac{\left(\left|standard\;device\;value-developed\;device\;value\right|\right)}{standard\;device\;value}\times 100$$

In this testing procedure, the researchers did not use decimals for calculating both humidity and temperature due to the use of the integer from the information shown in Table 2.

#### 2.5.1. Temperature and Humidity Sensor Calibration

The DHT22 sensor is used to measure both temperature and humidity. Temperature and humidity were recorded 36 times (20 s intervals for 12 min) within a controlled environment—a closed room without external wind and solar radiation influence. At the 6 min mark, an air conditioner was activated. The sensor’s readings were compared with the measurements obtained from a thermometer and hygrometer (Brannan^®^ Jumbo max min thermometer and hygrometer).

#### 2.5.2. Infrared Temperature Sensor Calibration

The GY-906-BAA MLX90614 sensor, designed for temperature measurement, was used in the research for body temperature measurement. To calibrate the sensor, temperature data were recorded 12 times (1 min intervals for 12 min) within a controlled environment—a closed room without external wind and solar radiation influence equipped with air conditioning. At the 6 min mark, an air conditioner was activated. This interval was chosen due to the reading time of the digital thermometer. Sensor readings were compared with measurements obtained from a digital thermometer (Omron^®^ model MC-246, Omron, Dalian, China). The infrared temperature sensor and digital thermometer were used to measure the temperature at the left axillary. The researchers chose not to use rectal measurements because these are impractical, making axillary measurements a better option for this study.

#### 2.5.3. Heart Rate Sensor Calibration

The MAX30102 sensor, designed for heart rate and oxygen measurement, was used in this research for heart rate measurement. To calibrate the sensor, heart rate data were recorded 12 times (1 min intervals for 12 min) within a controlled environment—a closed room without external wind and solar radiation influence equipped with air conditioning. At the 6 min mark, an air conditioner was activated. This interval was chosen due to the reading time of the pulse oximeter. The sensor attached to the left thumb fingertip and the probe of the pulse oximeter covered the left index fingertip. Sensor readings compared with measurements obtained from a pulse oximeter; Smiths Medical^®^ Spectro2 30: Digital Pulse Oximeter (Smiths Medical International Ltd., Colonial Way, Watford, Hertfordshire, WD24 4LG, UK), which is a commercial standard device for heart rate measurement (range 20–300 BPM (1 BPM increments), standard accuracy ±2 Arms, low perfusion accuracy ±3 Arms over 25–250 BPM range, less than 70% unspecified (Pulse amplitude < 1%. Tested using the industry-standard simulator)) [24]. In this study, heart rate device validity was not assessed by comparison with a gold standard such as an ECG or a heart rate monitor. However, the aim of this study is to preliminarily test for proving the device’s concept. In the future, the researchers will conduct tests against the gold standard, such as ECG, before human use to ensure true accuracy. Moreover, there are several studies which support the device validity using commercial medical standard devices [17,25].

## 3. Results

### 3.1. Sensors Calibration

#### 3.1.1. The DHT22 Temperature Measurements by Comparing with a Standard Thermometer

**Figure 5:**
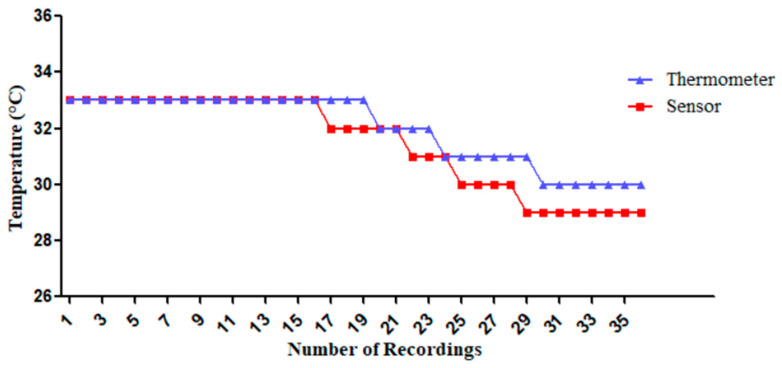
Comparison of ambient temperature measurements: thermometer vs. the DHT22 sensor before calibrating.

In this experiment, ambient temperatures were recorded 36 times (20 s intervals for 12 min, two sessions) using the DHT22 sensor for ambient temperature reading and the thermometer. The first 6 min occurred in a closed room without external wind and solar radiation influence. In the last 6 min of recording, the introduction of air conditioning caused an ambient temperature shift. The sensor’s performance closely matches the thermometer’s readings, with the former being slightly lower. Remarkably, the sensor measurements are more sensitive, showing faster decreases in temperature compared to the thermometer, as shown in Figure 5.

**Figure 6:**
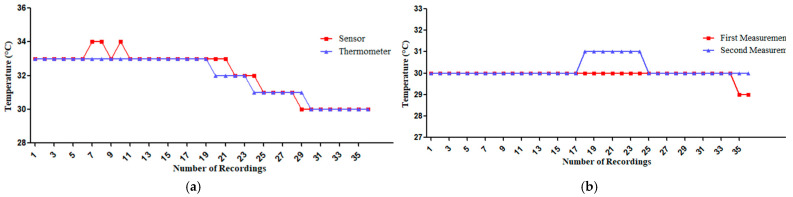
(**a**) Temperature measurements after sensor configuration: thermometer vs. adjusted the DHT22 sensor. (**b**) Comparison of first and second measurements of the DHT22 sensor for temperature measurements.

Then the researchers adjusted the sensor by increasing the value of ambient temperature readings by 0.9 °C in the Arduino IDE 2.3.2 which is the open-source Arduino software that is used to write code and upload it to the ESP32. However, the sensor detects temperature changes that are more delayed than the thermometer after the researchers adjusted it to match the thermometer measurements. The inaccuracies in the 7th, 8th, and 10th records were due to rounding up the readings. For the results from the DHT22 sensor for temperature measurements and the thermometer shown in Figure 6a and Table 5, the Shapiro–Wilk test revealed that the data distribution was not normal. Therefore, the researchers used the Spearman Correlation Coefficient which found that r = 0.923 with a significance of *p* < 0.001, indicating very high accuracy. Additionally, the Coefficient of Determination (R^2^) was 90.70, demonstrating a high degree of accuracy in the sensor’s performance after calibration. The sensor’s percent error remained below 5.00%, with was only at 3.23%.

However, for the results from the 1st and 2nd time of the DHT22 sensor for temperature measurements as shown in Figure 6b and Table 6, the unreliability observed in the records from the 17th to 25th times of the recording were evident in the measurements. The Shapiro–Wilk test indicated that the data distribution was not normal. Consequently, the researchers employed the Spearman Correlation Coefficient, which yielded a correlation coefficient of r = 0.489; however, this result was not statistically significant, indicating low reliability. Additionally, the Coefficient of Determination (R^2^) was 1.40, demonstrating a low degree of reliability in the sensor’s performance after calibration. However, the sensor’s percent error remained below 5.00%, with was only at 1.04%.

#### 3.1.2. The DHT22 Humidity Measurements by Comparing with a Standard Hygrometer

**Figure 7:**
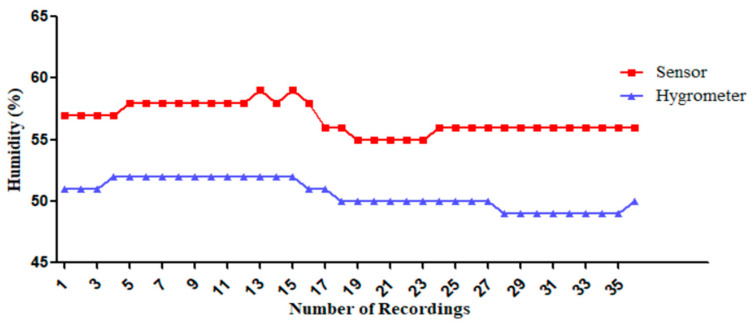
Comparison of humidity measurements: hygrometer vs. the DHT22 sensor.

In this experiment, humidity levels were recorded 36 times (20 s intervals for 12 min) using a hygrometer and a sensor in a closed room without external wind and solar radiation influence. In the last 6 min of recording, the introduction of air conditioning caused a significant drop in humidity as shown in Figure 7. The sensor’s measurements followed the same trend as the hygrometer’s, with a consistent difference of about 5%.

**Figure 8:**
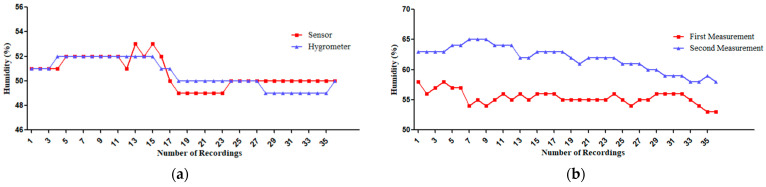
(**a**) Humidity measurements after sensor configuration: hygrometer vs. adjusted DHT22 sensor. (**b**) Comparison of first and second measurements of the DHT22 sensor for humidity measurements.

Therefore, the researchers adjusted the sensor by decreasing the value of ambient temperature measurements by 6.1% in the Arduino IDE. However, after adjusting the DHT22 sensor to match the standard hygrometer measurements, both the sensor and hygrometer generally followed the same trend, with some fluctuations in the measurements. The sensor tended to report slightly higher values compared to the hygrometer as shown in Figure 8a. For the results from the DHT22 sensor for humidity measurements and hygrometer as shown in Table 5, the Shapiro–Wilk test confirmed that the data distribution was not normal. Therefore, the researchers used the Spearman Correlation Coefficient which found that r = 0.774 with a significance of *p* < 0.001, indicating high accuracy. Additionally, the Coefficient of Determination (R^2^) was 63.90, demonstrating a moderate degree of accuracy in the sensor’s performance after calibration. The sensor’s percent error remained below 5.00%, with was only at 2.04%.

However, regarding the results from the first and second use of the DHT22 sensor for humidity measurements, as shown in Figure 8b and Table 6, the Shapiro–Wilk test revealed that the data distribution was not normal. Therefore, the researchers used the Spearman Correlation Coefficient which found that r = 0.181. However, this finding was not statistically significant, indicating low reliability. Additionally, the Coefficient of Determination (R^2^) was 8.30, demonstrating a low degree of reliability in the sensor’s performance after calibration. Moreover, the sensor’s percent error was also above 5.00%, reaching 11.51%.

#### 3.1.3. The GY-906-BAA MLX90614 Infrared Temperature Sensor Measurements by Comparing with a Standard Digital Thermometer

**Figure 9:**
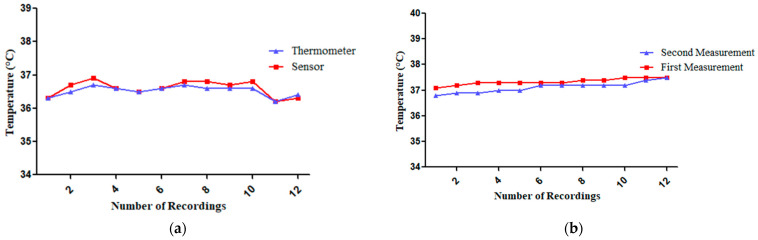
(**a**) Comparison of body temperature measurements: digital thermometer vs. the GY-906-BAA MLX90614 infrared temperature sensor. (**b**) Comparison of first and second measurements of the GY-906-BAA MLX90614 infrared temperature sensor.

In this experiment, body temperature measurements were recorded 12 times (1 min intervals for 12 min) using a standard digital thermometer and a sensor in an air-conditioned room. Both the sensor and the digital thermometer measured the temperature at the axillary. The digital thermometer and the sensor generally follow the same trend, with only a slight difference in their readings. Turning on the air conditioner at the sixth minute did not cause a noticeable change in body temperature as shown in Figure 9a. For the results as shown in Table 5 from the GY-906-BAA MLX90614 infrared temperature sensor and digital thermometer, the Shapiro–Wilk test confirmed that the data distribution was normal (*p* = 0.217). Therefore, the researchers used the Pearson Correlation Coefficient which found that r = 0.923 with a significance of *p* < 0.001, indicating very high accuracy. Additionally, the Coefficient of Determination (R^2^) was 85.10, demonstrating a high degree of accuracy in the sensor’s performance. The sensor’s percent error remained below 5.00%, with it only reaching 0.55%, without any adjustments.

Furthermore, for the results from the first and second use of the GY-906-BAA MLX90614 infrared temperature sensor, as shown in Figure 9b and Table 6, the Shapiro–Wilk test confirmed that the data distribution was normal. Therefore, the researchers used the Pearson Correlation Coefficient which found that r = 0.866 with a significance of *p* < 0.001, indicating high reliability. Additionally, the Coefficient of Determination (R^2^) was 75.00, demonstrating a high degree of reliability in the sensor’s performance. Moreover, the sensor’s percent error remained below 5.00%, with it only reaching 0.56%.

#### 3.1.4. The MAX30102 Heart Rate Sensor Measurements by Comparing with a Standard Pulse Oximeter

**Figure 10:**
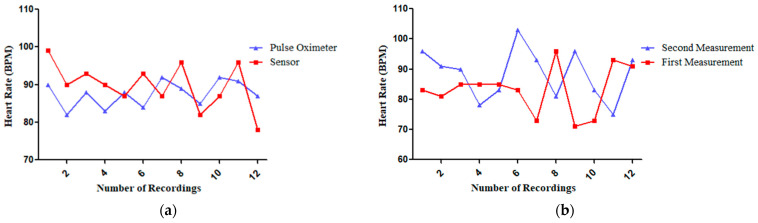
(**a**) Comparison of heart rate measurements: pulse oximeter vs. the MAX30102 heart rate sensor. (**b**) Comparison of first and second measurements of the MAX30102 heart rate sensor.

In this experiment, heart rate measurements were recorded 12 times (1 min intervals for 12 min) using a standard pulse oximeter and a sensor. The digital thermometer and the sensor have a similar trend, although the sensor’s graph shows a slight delay compared to the thermometer’s readings as shown in Figure 10a. For the results from the MAX30102 heart rate sensor and pulse oximeter as shown in Table 5, the Shapiro–Wilk test confirmed that the data distribution was normal. The researchers employed the Pearson Correlation Coefficient for their analysis, which yielded a correlation coefficient of r = 0.179. This result was not statistically significant, indicating no accuracy. Additionally, the Coefficient of Determination (R^2^) was 3.20, demonstrating a low degree of accuracy in the sensor’s performance. Unfortunately, the sensor exhibited an error exceeding the acceptable 5.00% threshold, averaging at 6.71% and reaching a maximum of 10.71%. These inconsistent differences—sometimes lower and sometimes higher than the pulse oximeter’s readings—made precise calibration impossible. Even after trying to adjust the sensor, the errors remained too high, so the researchers decided not to make further adjustments.

Furthermore, for the results from the first and second use of the MAX30102 heart rate sensor as shown in Figure 10b and Table 6, the Shapiro–Wilk test confirmed that the data distribution was normal. Therefore, the researchers used the Pearson Correlation Coefficient which found that r = 0.171 with no significance, indicating no reliability. Additionally, the Coefficient of Determination (R^2^) was 0.40, demonstrating a low degree of reliability in the sensor’s performance. Furthermore, the sensor exhibited a percent error exceeding 5.00%, specifically reaching 17.80%. 

### 3.2. Graphical User Interface

Our web application, developed using the Django framework, successfully bridges the gap between heat stroke monitoring and user-friendly data visualization. The website is used for displaying real-time data received from the device. Key features include intuitive data visualization, historical trend analysis, and educational content related to heat stroke prevention.

The website presents the risk of heat stroke based on sensor measurements. However, the accuracy and reliability of these sensors are very important. When the sensors provide accurate and reliably measurements, the website can more effectively calculate the risk of heat stroke, thereby enhancing prevention strategies.

**Figure 11:**
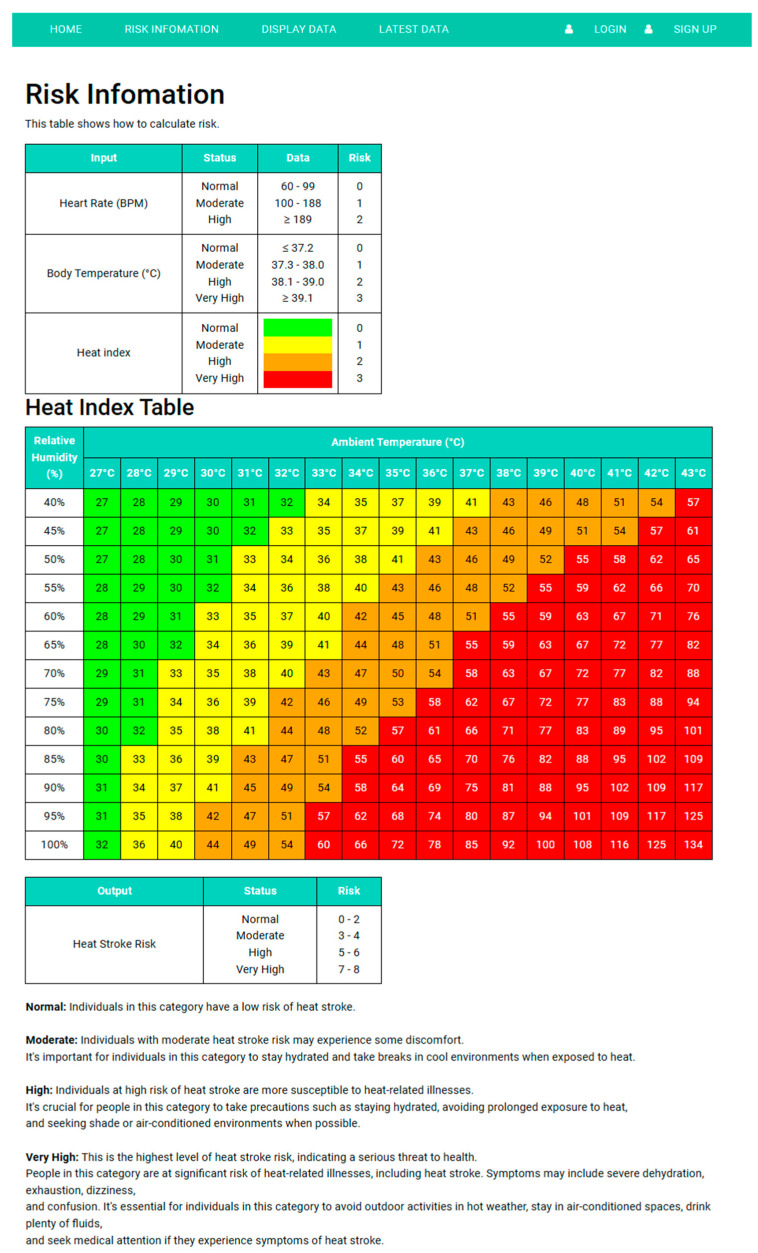
The risk information page.

The risk information page of the website provides users with essential tools and information to assess and manage heat stroke risks. This page features a heat stroke risk calculation table and a heat index table, both designed to help users understand the potential dangers associated with high temperatures and humidity. Additionally, detailed text at the bottom of the page offers instructions on definitions of key terms, precautions for different risk levels, signs of heat-related illnesses, and first aid recommendations as shown in Figure 11.

**Figure 12:**
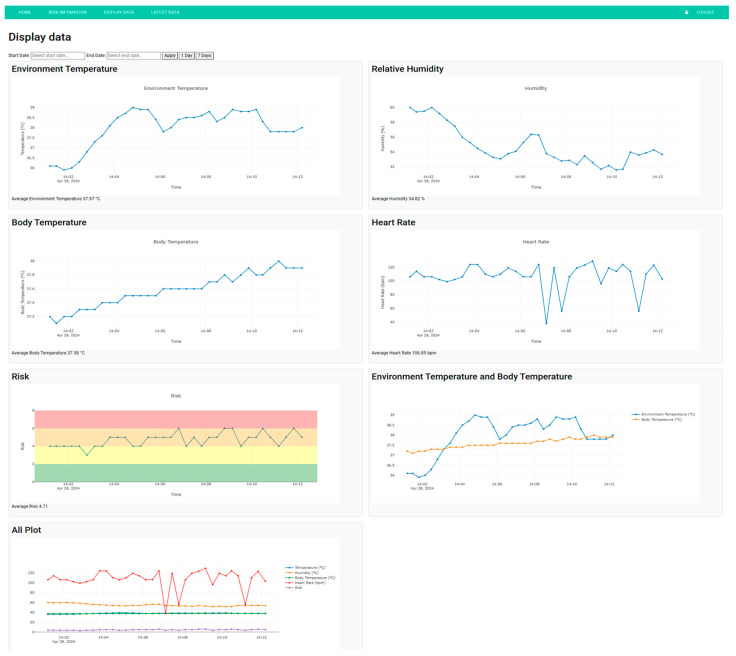
The display data page.

The data display page of the website provides users with a comprehensive view of various health and environmental metrics over time. Users can select specific dates to visualize data. These include individual graphs for ambient temperature, relative humidity, body temperature, and heart rate. Additionally, a combined graph illustrates the relationship between ambient temperature and body temperature, offering deeper insights into how these variables interact. A risk graph, color-coded to indicate different risk levels, helps users quickly assess potential health threats. Finally, an all-encompassing plot integrates all the data into a single graph, providing a holistic view of the monitored parameters, as shown in Figure 12.

It is important to note that the figures presented in this section are illustrative and not based on data collected from the experiment. They are designed to demonstrate the functionality and potential applications of the website’s data visualization capabilities. These figures serve as examples to help users understand how the system can be used to monitor and analyze health and environmental metrics.

## 4. Discussion

Our heat stroke detection device prototype can integrate sensors to monitor environmental conditions and physiological parameters. The device calculates the heat index and triggers alerts when it surpasses a threshold. It can be used for more than 24 h on a single charge from a 10,000 mAh power supply. The device sends data using Wi-Fi, making it suitable for long-distance sports like biking and marathon running when used with a mobile hotspot. Additionally, the researchers developed a web-based monitoring dashboard for real-time data visualization. However, a critical challenge remains, where not all sensors meet the accuracy and reliability requirements of high accuracy and reliability and less than 5% error.

Moreover, the DHT22 sensor which is widespread for tracking ambient temperature and humidity as shown in Son, Ramli, and Aziz (2021) [6] provides highly accurate temperature and humidity readings, as confirmed by calibration results using the Correlation Coefficient and percentage error. To further enhance accuracy and reliability, future calibration should focus on adopting a standard calibration method such as the salt test [26], which involves comparing a hygrometer reading with saturated salt solutions at a certain temperature. This will ensure, especially, the reliability of both temperature and humidity data from the DHT22 sensor.

Furthermore, the GY-906-BAA MLX90614 infrared body temperature sensor used in Chen, Lin, Lan, and Hsu (2017) [5] has high accuracy, as confirmed by calibration results using the Correlation Coefficient and percentage error. However, there is potential for further improvement. By adopting a more precise method for calculating body temperature, as outlined in Gammel, J (2016) [27], which involves using a formula to calculate body temperature from a specific spot on the human body combined with ambient temperature, the accuracy and the reliability of the GY-906-BAA MLX90614 sensor can be enhanced even further.

In future tests of accuracy and reliability, it is essential to control various factors more rigorously. For example, conducting experiments in a room where temperature and humidity can be controlled will ensure that the test results, especially those related to reliability, yield accurate values when measurements are repeated.

Moreover, the website provides information about heat stroke risk, explaining how it is calculated and what actions to take when at risk. It also features data presented in graphs, capable of collecting large amounts of data, allowing users to observe trends, get an overview, and analyze historical data by selecting start and end dates. Each measured value, such as body temperature, ambient temperature, relative humidity, and heart rate, is displayed in separate graphs for clarity. The website can track data for multiple users. It is free to use with no restrictions and is accessible on any platform. While current sensors may not be perfectly accurate and reliable, the researchers are actively working to improve their accuracy and reliability. In the future, we hope that these advancements will enable our website to serve as an invaluable tool for the pre-assessment and prevention of heat stroke. However, the users should undergo repeated medical checks to ensure accuracy in preventing the dangers of heat stroke. Additionally, it is important to note that presently the website notifications are not in real-time, experiencing a delay of approximately one minute.

This research developed only a heat stroke prevention website; however, to enhance user experience and data interpretation, future integration of mobile apps could significantly improve data visualization. Unlike websites, which often face limitations such as screen size, resolution, and interaction constraints, mobile apps provide a more engaging and accessible platform. Furthermore, mobile notifications can deliver real-time updates, ensuring users receive timely insights to help them stay informed and proactive in preventing heat-related issues.

The device has several limitations that impact on its accuracy and reliability. The use of Infrared Thermography (IRT) for measuring skin temperature has a limited association with core body temperature. Additionally, measuring skin temperature at only one location does not account for variations across different body parts, potentially leading to inaccuracies. Moreover, the skin temperature measured at the axillary reflect the core body temperature [7]; however, in this study, the body temperature did not evaluate at the rectal or blood temperature. Another limitation of this study is the validation of heart rate measurements. As the research of Stracina et al. (2022) [28] shows, the ECG is the gold standard for heart rate validation. However, in this study, the researchers validated MAX30102 with a commercial standard medical device which is practical and has been supported by similar studies [17,25]. Although this research did not test against the gold standard, the preliminary tests indicate a direction of accuracy to prove the device’s concept. However, in the future, the researchers will conduct tests against the gold standard, such as ECG, before human use to ensure true accuracy.

Although a 10,000 mAh power supply allows for more than 24 h of use, this might not be sufficient for longer events or multi-day activities without access to charging facilities. Moreover, it is a prototype, it may not be as comfortable to wear for extended periods, and further refinements are needed to improve its ergonomics and user comfort. In addition, the Wi-Fi dependency for data transmission could be a limitation in areas with poor connectivity or during activities where maintaining a stable connection is challenging. This reliance on a mobile hotspot might also be inconvenient for users. Only this developed device is required for the athlete; however, a mobile phone may be necessary to provide an internet connection via a mobile hotspot. A mobile phone will not be needed if the location already has an established internet connection. Lastly, the web-based monitoring dashboard, although a great feature, requires users to have access to a device with internet connectivity, which might not always be practical during outdoor activities.

Furthermore, factors such as age, gender, and physical fitness can significantly impact heat tolerance. In addition, the difference of environmental conditions can face varying levels of risk. At a normal status level, people tend to be at low risk, such as people in air-conditioned rooms. At a moderate status, people tend to be in a well-ventilated room with a fan on. Those in high status areas tend to be outdoors where there may be some shade or people who are playing sports in a ventilated place but the temperature outside is high causing the temperature inside the body to rise accordingly. People with very high status tend to have activities outdoors in high temperatures such as engaging in sports with no shade in the daytime. Moreover, groups of patients in Mimish (2012) [29] have shown different ranges of vital signs based on age and gender. Additionally, solar radiation affects heat tolerance. Research by H. Otani et al. (2016) [30] has shown that solar radiation affects mean skin temperature at four sites (chest, upper arm, thigh, and calf). The highest level of solar radiation resulted in much higher skin temperatures compared to lower levels. Furthermore, the difference between core and skin temperatures was smaller under the highest solar radiation, indicating a reduced ability to dissipate heat. These findings highlight the significant impact of solar radiation on the body’s ability to regulate temperature during outdoor activities.

Additionally, the device needs to be user-friendly. Making the device lightweight, easy to wear, and comfortable—by using ergonomic shapes and adjustable straps—will help people use it more easily without interfering with their daily activities.

In the future, it is imperative that advancements in sensor systems are pursued to significantly enhance their accuracy and reliability across various applications. Furthermore, systematic strengths must be undertaken to collect comprehensive data regarding both the accuracy and reliability of these systems in athletes while outdoor exercising. It is essential that rigorous testing be conducted to assess the effectiveness of these sensors in detecting and preventing heat stroke among athletes during training sessions and actual competitions, thereby providing a thorough evaluation of the tools’ true performance. This research is only a pilot study which investigates the feasibility and safety of the sensors and it can introduce bias; however, the researchers suggest that in the future, other studies should increase the sample size, use blind or double-blind study designs, and ensure the random selection of participants to enhance the reliability and validity of research findings.

## 5. Conclusions

This research has developed a heat stroke warning system prototype for athletes that measures values from sensors, including DHT22, GY-906-BAA MLX90614 and MAX30102. The system calculates the risk of heat stroke and notifies users via a buzzer. The obtained data are recorded, stored in a database, and displayed on the website which provides alert notifications when the risk of heat stroke is high. The website displays the data as a graph of the body temperature, ambient temperature, relative humidity, heart rate, and risk of heat stroke which is free to use with no restrictions and is accessible on any platform, making it especially useful for those in tropical zones or engaging in outdoor workouts.

The findings indicate that the DHT22 sensor demonstrates high accuracy in ambient temperature measurements, alongside in humidity measurements. However, it showed no significant reliability in ambient temperature measurements, as well as in humidity. Conversely, the GY-906-BAA MLX90614 sensor exhibited both high accuracy and reliability in body temperature measurements. In contrast, the MAX30102 sensor lacked significant accuracy and showed no significant reliability in heart rate measurements. Therefore, enhancing both the accuracy and reliability of these sensors is important for effectively monitoring and preventing heat stroke in future applications.

## Figures and Tables

**Table 1:** Input data and heat stroke risk.

Input	Status	Data	Heat Stroke Risk
Heart Rate (BPM)	Normal	60–99	0
	Moderate	100–188	1
	High	≥189	2
Body Temperature (°C)	Normal	≤37.2	0
	Moderate	37.3–38.0	1
	High	38.1–39.0	2
	Very High	≥39.1	3

**Table 2:** Heat index values in Celsius.

Temperature (°C)																		
Relative Humidity (%)		27	28	29	30	31	32	33	34	35	36	37	38	39	40	41	42	43
	40	27	28	29	30	31	32	34	35	37	39	41	43	46	48	51	54	57
	45	27	28	29	30	32	33	35	37	39	41	43	46	49	51	54	57	61
	50	27	28	30	31	33	34	36	38	41	43	46	49	52	55	58	62	65
	55	28	29	30	32	34	36	38	40	43	46	48	52	55	59	62	66	70
	60	28	29	31	33	35	37	40	42	45	48	51	55	59	63	67	71	76
	65	28	30	32	34	36	39	41	44	48	51	55	59	63	67	72	77	82
	70	29	31	33	35	38	40	43	47	50	54	58	63	67	72	77	82	88
	75	29	31	34	36	39	42	46	49	53	58	62	67	72	77	83	88	94
	80	30	32	35	38	41	44	48	52	57	61	66	71	77	83	89	95	101
	85	30	33	36	39	43	47	51	55	60	65	70	76	82	88	95	102	109
	90	31	34	37	41	45	49	54	58	64	69	75	81	88	95	102	109	117
	95	31	35	38	42	47	51	57	62	68	74	80	87	94	101	109	117	125
	100	32	36	40	44	49	54	60	66	72	78	85	92	100	108	116	125	134

Normal status 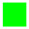, Moderate status 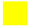, High status 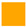, Very High status 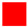.

**Table 3:** Heat index status and heat stroke risk.

Heat Index (°C)	Status Color	Status	Heat Stroke Risk
27–32		Normal	0–2
33–41		Moderate	3–5
42–54		High	5–6
≥55		Very High	7–8

The status colors enhance users’ comprehension on websites.

**Table 4:** Heat stroke risk status.

Output	Status	Heat Stroke Risk
Heat Stroke Risk Status	Normal	0
	Moderate	1
	High	2
	Very High	3

**Table 5:** Presents the results from developed device and standard device for ambient temperature, relative humidity, body temperature, and heart rate measurements.

Parameters		Developed Device Measurement	Standard Device Measurement
Ambient Temperature (°C)	Mean (SD)	32.16 (1.49)	31.97 (1.23)
	r	0.923 *	
	%Error	0.59	
	R^2^	90.70	
Relative Humidity (%)	Mean (SD)	50.59 (1.11)	50.58 (1.18)
	r	0.774 *	
	%Error	0.02	
	R^2^	63.90	
Body Temperature (°C)	Mean (SD)	36.60 (0.23)	36.53 (0.15)
	r	0.923 *	
	%Error	0.19	
	R^2^	85.10	
Heart rate (BPM)	Mean (SD)	89.83 (6.07)	87.58 (3.45)
	r	0.179	
	%Error	2.57	
	R^2^	3.20	

* Significant at *p* < 0.01 (Sig. (two-tailed)).

**Table 6:** The results from the first and second use of the developed device for ambient temperature, relative humidity, body temperature, and heart rate measurements.

Parameters		Developed Device Measurement	
		1st Time	2nd Time
Ambient Temperature (°C)	Mean (SD)	29.84 (0.20)	30.15 (0.28)
	r	0.489	
	%Error	1.04	
	R^2^	1.40	
Relative Humidity (%)	Mean (SD)	55.41 (1.21)	61.79 (1.98)
	r	0.185	
	%Error	11.51	
	R^2^	8.30	
Body Temperature (°C)	Mean (SD)	37.34 (0.12)	37.13 (0.21)
	r	0.866 *	
	%Error	0.56	
	R^2^	75.00	
Heart rate (BPM)	Mean (SD)	83.25 (7.93)	88.50 (8.43)
	r	0.171	
	%Error	17.80	
	R^2^	0.40	

* Significant at *p* < 0.01 (Sig. (two-tailed)).

## Data Availability

The raw data supporting the conclusions of this article will be made available by the authors on request.
